# Experimental Models in Penile Transplantation: Translational Insights and Relevance to Clinical Application

**DOI:** 10.7759/cureus.74258

**Published:** 2024-11-22

**Authors:** Naga Anvesh Kodali, Ramu Janarthanan, Bedreddin Sazoglu, Zeynep Demir, Omer Faruk Dirican, Yalcin Kulahci, Fatih Zor, Vijay S Gorantla

**Affiliations:** 1 Department of Surgery, Wake Forest School of Medicine, Winston-Salem, USA; 2 Department of Plastic and Reconstructive Surgery, Amrita Institute of Medical Sciences and Research Centre, Amrita Vishwa Vidyapeetham, Kochi, IND; 3 Department of Plastic and Reconstructive Surgery, Indiana University School of Medicine, Indianapolis, USA

**Keywords:** experimental models, penile transplantation, penis, vascularized composite allotransplantation, vca

## Abstract

Animal research is an essential contributor to the medical achievements of the last century. The first step of studying a disease in animals is the development of a model which is relevant to the clinical situation in humans. Thus, a good animal model is the sine qua non of the experimental research. This review aims to assess the contemporary literature on animal models for penile transplantation, examining their applicability and significance in the context of clinical scenarios. We also revisit, evaluate, and emphasize the interesting and important findings of certain animal models to bring the reader up to date from the perspective of allotransplantation. Their current and future clinical applicability and feasibility have been discussed, shedding light on worldwide experience in Vascularized Composite Allotransplantation (VCA).

## Introduction and background

Introduction

Almost two decades ago, the new era in clinical reconstructive transplantation began with the first successful hand transplantation in 1998 [1]. Following this breakthrough, the first face transplantation was performed in 2005 [2], and the clinical applications of various vascularized composite allotransplants have continued to increase. One of the most recent and interesting vascularized composite allotransplantation (VCA) attempts includes penile or genitourinary transplantation. To date, five cases of penile allotransplantation [3-9] have been performed. Penile transplantation, considered a life-enhancing procedure, remains a subject of ongoing medical and ethical debates in the scientific community, especially as psychological aspects play a significant role in this field of transplantation compared to other VCAs [7, 10-12]. This study aims to review the reported animal models [11, 13-21] of penile transplantation and discuss their relevance to the clinical situation and effectiveness in mimicking human applications.

Methods

The methodology used to write this review article involved conducting a comprehensive PubMed search between January 2000 and October 2023. The search included keywords such as "Penile Transplantation," "Vascularized Composite Allotransplantation," "VCA," and "Penis." ​ The authors assessed contemporary literature on animal models for penile transplantation, examining their applicability and significance in clinical scenarios. ​ They revisited, evaluated, and emphasized interesting and important findings from certain animal models to provide an up-to-date perspective on allotransplantation. ​ The review also discusses the current and future clinical applicability and feasibility of these models, shedding light on worldwide experiences in VCA. ​

## Review

Clinical cases of penile transplantation

As of the date of writing this article, five cases of penile transplantation have been performed worldwide [3-5, 7-9] (Table 1).

**Table 1:** Clinical cases of penile transplantation

The surgical team and year performed	Cause	Follow-up	Clinical outcomes
Hu et al., 2006, China [4-6]	A 44-year-old male with a traumatic penile defect.	14 day	Due to severe psychological problems experienced by the recipient and his wife, the transplanted penis was cut off on post-transplant day 14.
Merwe et al., 2014, South Africa [6-8, 10, 12]	A 19-year-old male who lost his penis in 2012 due to complications of ritual circumcision. After 2 years of his penile loss, the patient received a penile allograft in 2014, at the age of 21.	> 8 years	The authors reported the full sexual and urinary function of the patient 3.5 months after transplantation. However, the patient lost half of his graft due to rejection 32 months after transplantation. This rejection was successfully reversed, and the patient also had skin grafts [6, 8]. Even his partner is pregnant, according to Merwe et al. [8], however, the child was stillborn at term.
Cetrulo et al., 2016, USA [3, 6]	A 64-year-old male with subtotal penectomy due to penile cancer	> 5 years	The authors reported recovery of the partial sensation of the penile shaft and spontaneous tumescence in the penis at the 7th month post-transplant [3]. Patient's overall satisfaction related to health, self-image, and future optimism is improved. The patient is doing good even after 5 years [6].
Merwe et al., 2017, South Africa [6, 8]	A 40-year-old male who lost his penis at the age of 22 (in 1999), due to complications from ritual circumcision.	> 4 years	In 2021, Merwe et al. [8] reported that the patient had regained his sexual function and successfully entered into a sexual relationship after his transplantation. However, Lopez et al. reported that the flap was removed 54 months after the transplantation due to rejection and necrosis [6].
Richard J Redett 3rd et al., 2018, USA [6, 9]	An injured veteran who sustained traumatic penile loss, along with above-knee amputation of both legs, substantial tissue loss in the lower abdominal wall, bilateral orchiectomy, and loss of the scrotum. The transplantation of the penis, scrotum, and lower abdominal wall was performed on a young, closely age-matched donor	>3 years	The one-year follow-up was reported by Redett et al., detailing that the patient had near-normal erections, could achieve orgasms, and had normal sensations at the shaft and tip of the transplanted penis [9]. Lopez et al. reported that the patient is doing well 45 months after the transplantation [6].

In 2006, Hu W, et al. performed the first ever human penile transplant using a microsurgical procedure in China on a 44-year-old male who lost his penis due to trauma. They performed anastomoses of the deep dorsal vein, dorsal artery, and superficial dorsal vein. The patient did not have any problems during the initial post-operative period, however, the penile tissue experienced severe venous congestion along with necrosis of the skin. Along with these issues, the patient and his wife came up with some psychological issues on the 14th day following the transplant, which ultimately led to the removal of the graft [4, 5].

In 2014, Van der Merwe et al. performed the second penile VCA transplant, in South Africa, on a patient who had lost his penis during a ritual circumcision. They used deep inferior epigastric vessels as the recipient's vessel. Despite encountering hematoma and urethrocutaneous fistula during the immediate postoperative period, the patient’s long-term outcome was satisfactory. By day 100 after the transplant, he had achieved good erectile function and reported a normal desire for sex. He also reported satisfactory intercourse two years after the transplantation [7, 8, 12]. However, at 32 months post-transplantation, the patient experienced rejection, resulting in the loss of half of the graft. This rejection was successfully reversed, and the patient underwent skin grafts [6, 8]. Additionally, he was able to impregnate his partner, although the child was stillborn at term [8].

In 2016, Cetrulo et al. performed the world’s third penile transplantation, marking the first attempt at a penile transplant in the United States [3, 6, 22]. The recipient had a history of penile cancer, leading to subtotal penectomy. The surgery involved a microsurgical procedure performed by two surgical teams. The authors anastomosed the cavernosal arteries and the deep dorsal vein. They used a vein graft for due sclerotic recipient dorsal arteries, anastomosed end-to-side to the right femoral artery and end-to-end to the right dorsal penile artery [3]. The initial post-operative period necessitated two additional surgeries on postoperative days 2 and 13 for the hematoma evacuation and skin debridement due to eschar, respectively. Subsequently, the patient reported a good urinary stream, erectile function, and sensation at the penile shaft 6 months after the procedure. The patient experienced satisfactory results with no complaints of fistulae or strictures at 66 months following the transplantation [22].

In 2017, the same South African team, led by van der Merwe et al., performed the fourth penile transplant, following their earlier success with the first transplant [6-8, 12]. They used deep inferior epigastric vessels as the recipient's vessel. The authors published their follow-up in 2021, reporting that the patient had regained his sexual function and successfully entered into a sexual relationship after his transplantation [8]. However, the transplanted flap was removed due to rejection and necrosis 4 years and 6 months after the transplant surgery [6].

The fifth and most recent penile transplantation was performed by Richard J Redett 3rd et al. in 2018. This procedure involved the transplantation of the entire penis, scrotum, and the lower abdominal wall [9]. They used deep inferior epigastric vessels as the recipient's vessel. The recipient had injuries in multiple regions, including severe loss of tissue in the lower abdominal wall, an above-knee amputation of both legs, and bilateral traumatic orchiectomy with loss of the scrotum. The surgical team successfully transplanted the entire penis, scrotum, and lower abdominal wall. The patient reported a normal sensation at the penile shaft and was able to achieve good erection, regular urination, and satisfactory orgasms [9].

Despite the expansion in the field of VCA, the number of penile transplants performed over these years has been limited. The surgical technique and surgery-related complications still need to be addressed through pre-clinical animal studies for optimal clinical outcomes. The unique and complex anatomical and physiological nature of penile tissue sets it apart from other VCAs. The probability of post-operative rejection, and ethical dilemmas involved in this sensitive area of allotransplantation remain very high, necessitating further research in this domain [23].

Experimental models of penile transplantation

Penile transplantation represents a novel clinical application of VCA. Despite there being five cases of penile transplantation performed to date [6, 22], ethical and medical controversies persist surrounding this procedure. Additionally, assessing the psychological facets of penile transplantation presents a substantial challenge. One critical approach to addressing these debates is by collecting scientific data from experimental models. Various animal models have been studied to understand the feasibility and outcomes of penile transplantation, each contributing unique insights into the procedure. ​Animal models of replantation and autotransplantation of the penis were studied by Akyurek et al. [13], Karamursel et al. [16], Seyam et al. [19], and Raney et al. [18] (Table 2). The first case of successful clinical penile replantation dates back more than 50 years ago and was performed by Tuerk et al. in 1971 [24]. In 1977, Tamai et al. successfully replanted an entirely severed penis and scrotum [25].

**Table 2:** Animal models of penile transplantation and their clinical relevance * This section presents the properties of the currently published models, which are closely related to future translational studies. However, even with necessary anatomical repairs, none of them reflect the real clinical scenario in humans. (-) the model currently lacks this property, (+) the model already possesses this property, (±) not evaluated but briefly discussed.

Type of transplantation and Model name		Species	Author	The suitability of the model for translation from animal models to clinical studies*				
				Arterial anastomosis	Urethral repair	Nerve repair/ Evaluation of sensation	Evaluation of Erection	Immunology related studies
Murine Models								
Auto-transplantation models	Penile flap (orthotopically replaced in situ/ heterotopic)	Rat	Akyurek et al., 2005 [13].	+	-	+ / -	-	-
	Penis replantation model (orthotopic/ heterotopic)	Rat	Karamursel et al., 2005 [16].	+	-	- / -	-	-
	Penile autotransplantation (orthotopic)	Rat	Seyam et al., 2013 [19].	-	+	+ / -	-	-
Allogeneic Models	Nonvascularized allotransplantation (heterotopic)	Rat	Koga et al., 2003 [17].	-	-	- / -	-	+
Allogeneic/ Syngeneic Models	Vascularized transplantation (heterotopic)	Rat	Fidder et al., 2020, 2019 [14, 15].	+	-	+ / -	-	+
	Vascularized transplantation (orthotopic)	Rat	Sonmez et al., 2009 [20].	+	-	+ / +	-	+
Syngeneic Models	Multivisceral transplantation of pelvic organs (orthotopic/ heterotopic)	Rat	Galvao et al., 2023 [11].	+	-	+ / -	-	-
Canine Models								
Allogeneic Models	Vascularized allotransplantation (orthotopic)	Dog	Zhao et al., 2016 [21].	+	+	+ / -	-	+
Syngeneic Models	Dog penile replantation model without vascular anastomosis (orthotopic)	Dog	Raney et al., 1975 [18].	-	-	-	±	-

Murine models of penile transplantation

Autologous Transplantation Models of Penis

Penile flap (orthotopically replaced in situ/heterotopic): Akyurek et al. described the penile composite tissue flap based on the internal pudendal vessels in rats in 2005. In their study, they harvested the penile flap, including penile body and preputial skin, as an island flap and re-inserted as an in situ in 10 rats. Additionally, they performed 10 heterotopic penile transplantations to the groin region via anastomoses between internal pudendal and superficial epigastric vessels [13]. Pudendal nerve coaptation was not performed in this model, as the aim of the study was to demonstrate the operational feasibility of penile transplantation. The authors demonstrated the viability of the composite penile flap following transplantation. However, this model did not report functional outcomes regarding urethral repair and erectile function, highlighting a gap in understanding the full clinical relevance of the procedure. ​

Penile replantation model (orthotopic/ heterotopic): A new microsurgical training model of rat penile replantation was reported by Karamursel et al. in 2005. The rat penis, including two corpora cavernosa, corpus spongiosum, prepuce, and glans was harvested based on the internal pudendal artery and internal pudendal vein. They transferred the penile flap to two different recipient areas. The first was the right thigh, where the femoral artery and vein served as the recipient's vessels. The second area was the original place, and the penile flap was anastomosed to the saphenous artery and vein. Similar to Akyurek et al., this model did not include pudendal nerve coaptation, thus limiting its clinical and functional relevance [16].

Penile autotransplantation (orthotopic): Seyam et al. introduced a penile autotransplantation model in rats in 2013. Although the authors performed the anastomoses of the tunica albuginea, urethra, dorsal vein, and dorsal nerves, they did not perform the dorsal arterial anastomosis. ​Consequently, the primary weakness of this model lies in the absence of arterial supply, resulting in poor long-term viability of the distal urethra, glans, and penile skin, as their vascularity relies solely on venous supply. As acknowledged by the authors, their suggested model resulted in poor long-term viability of certain penile tissues, emphasizing the importance of arterial supply for successful transplantation. This model is only suitable for evaluating the viability and function of the corpora cavernosa [19].

Syngeneic/Allogeneic and/or Heterotopic/Orthotopic Transplantation Models of Penis

Nonvascularized penile allotransplantation model (heterotopic model): Koga et al. published a nonvascularized allogeneic penile transplantation model in Brown-Norway donors and Lewis recipient rats in 2003 [17]. The allogeneic penis was covered with omentum and awaited its vascularization. However, the penis was harvested as a graft without its vascular pedicle, which was the primary drawback of this model. Although the authors concluded that it could be transferred based on omentum, its experimental and clinical applicability is not reproducible. Another drawback is that this model required two laparotomies for placing the graft in the omentum and harvesting the graft. Subsequently, after the graft has achieved vascularization, the omentum and penile graft become intertwined, making their separation extremely challenging [17]. As a conclusion, the need for two laparotomies and the difficulty in separating the omentum from the graft further limited its clinical practicality. ​

Vascularized Penile Allotransplantation Models (Syngeneic/Allogeneic and/or Heterotopic/orthotopic Models)

Allogeneic/syngeneic orthotopic model: The first neurovascular penile allotransplantation model was introduced by Sonmez et al. [20]. The penile vascular pedicle was utilized for immediate microsurgical revascularization, which made it significantly different from Koga et al.’s model. However, from the vascular pedicle perspective, this model also exhibits some unique differences from other penile transplantation models [13, 16, 19]. The arterial supply of the penis was provided by the end-to-end anastomosis of the corpus spongiosum to the saphenous artery. In contrast to pudendal artery-pedicle penile transplantation models [13, 16], this approach significantly shortened the operation time and ischemia. The dorsal penile vein was anastomosed to the saphenous vein, and the dorsal penile nerve was coapted to the lateral cutaneous femoral nerve for sensory innervation. Cyclosporine A was used as an immunosuppressive drug. At the hundredth post-transplant day, histologic and microangiographic evaluation of the penile tissues revealed complete viability of the graft. Somatosensory evoked potentials (SSEP) confirmed the sensory recovery of the flap. The authors reported over 200 days long time allograft survival. This model is the first to demonstrate sensory re-innervation of the transplanted penis and long-term graft survival, lacking urethral and erectile functional outcomes ​[20].

Allogeneic/syngeneic heterotopic model: Fidder et al. addressed the lack of understanding regarding the immunological aspects of penile transplants, which has hindered their application in urogenital reconstruction [14, 15]. They introduced a new rat heterotopic penile transplant model, incorporating preputial skin, to establish a rejection classification for urogenital tissue transplants. The study involved syngeneic and allogeneic penile transplantations in rats, with all syngeneic and tacrolimus-treated grafts surviving. The rat penile graft, along with preputial skin, was based on the internal pudendal artery and dorsal penile vein. It utilized a nonsuture cuff technique for anastomosis with the recipient's superficial epigastric and femoral vessels. Seventy-seven penile transplantations were conducted, demonstrating that the graft design provides a suitable caliber and length of vessels at the penis radix. Unlike the model published by Sonmez et al. [20], optimal graft perfusion was ensured through the anastomosis of the dorsal penile vein and internal pudendal arteries. The nonsuture cuff technique enabled successful microvascular anastomosis by a single surgeon, with an average overall operative time of 2.5 hours. Syngeneic transplants exhibited long-term graft survival (>30 days). The researchers established a robust murine model with good vascular perfusion to the penile tissue, enabling the study of the unique immunobiology of male genitourinary allotransplantation. The allogeneic graft rejection exhibited a four-stage clinical progression, with untreated grafts experiencing epidermal sloughing at postoperative day 7 and complete rejection between days 14 and 16. The heterotopic inset also facilitates visual monitoring of graft viability, with the native penis serving as an optimal control. The researchers developed a specific four-grade rejection classification, akin to the 2007 Banff Criteria, focusing on graft skin and urethral lining as primary rejection targets [14, 15]. In summary, their study established a rejection classification for urogenital tissue transplants and demonstrated good vascular perfusion, providing a robust model for studying immunobiology.​

Whole Pelvic Floor Transplantation Model (Syngeneic and Orthotopic)

In 2023, Galvao et al. demonstrated the feasibility of a microsurgical technique for multivisceral transplantation of pelvic organs, including the pelvic floor, in rats. The donor operation involved a perineal and abdominal incision, allowing en bloc removal of the graft containing various pelvic organs. The recipient's operation included aortic and vena cava anastomoses, as well as connections for the ureter, rectum, and pudendal nerves. The pelvic floor was repositioned orthotopically or heterotopically. Out of seven orthotopic and four heterotopic transplantations, an 81.82% early survival rate was achieved, with two animals succumbing to technical failure [11]. This model opens up new possibilities for the transplantation of the pelvic floor as a whole unit and shows a high early-survival rate despite some technical failures. ​

Canine models of penile transplantation

Dog Penile Replantation Model Without Vascular Anastomosis (Orthotopic Model)

Raney et al. discussed a penile replantation model in dogs in 1975, addressing the challenges associated with amputated penises and sharing their experience with intentional transection and reanastomosis in canine subjects [18]. They performed penile transections on 10 mongrel dogs, reanastomosing the penis at various time intervals without vascular anastomosis. The authors reiterated that the presented model is not a microsurgical anastomosis model; they attached the amputated distal penile tissue (glans penis) layer by layer using chromic catgut. Results showed initial pallor and congestion, followed by sloughing of the distal portion, which eventually granulated and formed new skin. Histological examination revealed some tissue damage, with variations in healing among dogs. Complications such as distal urethral slough, urethrocutaneous fistula, and urethral stricture were discussed. They suggested that future efforts at the reanastomosis of amputated penises could be aided by microsurgical techniques. The study also discussed the canine penile anatomy in relation to erectile tissue development. Overall, these initial findings by Raney et al. encouraged future authors to develop microsurgical models of penile transplantation Their findings encouraged the need for the development of microsurgical models in large animals [18].

Dog Penile Allotransplantation Model (Orthotopic Model)

In 2016, Zhao et al. published a penile allotransplantation model involving 20 Beagle dogs. The distal part (3 cm) of the penis was resected in recipient dogs and repaired with the penis harvested from donor animals. In this model, dorsal vein, artery, and nerve anastomosis were performed between the donor and the recipient, in addition to the repair of erectile tissues (cavernous and spongious bodies) and the urethra. Out of the 20 transplanted penises, 12 survived under the immunosuppressive protocol. The transplanted penises were resected on post-transplant day 14th for pathological examination, revealing normal penile structures without interstitial edema [21]. The nerve and vessel histology was normal, and the authors also reported normal urination of the transplanted penis without urethrostenosis. This large animal model is more similar to human allotransplantation setting than rat models [13, 16, 19]. This model showed normal penile structures and urination post-transplant, but the short follow-up and lack of erectile function evaluation were significant drawbacks for clinical translation [21].

Clinical challenges of penile transplantation

Unlike other VCA, many issues remain to be resolved before the routine clinical application of penile allotransplantation. The current literature reveals numerous unknown areas about penile transplantation. The explantation of the penile graft in two out of the five clinical cases indicates that the long-term viability of the graft remains a challenge to this date. This challenge is attributed to complications, including hematoma formation, vascular congestion, necrosis of the graft tissue, and rejection [3, 6, 7]. These issues need to be addressed by animal models, although we are aware that the translation of these animal models to clinical application is a challenge in itself.

Registry, Donation, and Organ Procurement-Related Issues

The national or international registry systems for Solid Organ Transplantation (SOT) and VCA are essential for registering new cases and analyzing transplantation outcomes. A penile allograft consists of various tissues such as corpus cavernosum, urethra, corpus spongiosum, vessels, nerves, and skin; thus, it is also regarded as VCA and can be registered under the VCA registry system [26-28]. Donation of solid organs is more common than VCA, and families may be more willing to consent to such donations compared to those involving vascularized composite tissue. However, from some other perspectives such as marriage, religious, psychological, social, and personification, the donation of the penis may present greater challenges for the family [26, 28-31].

Organ procurement: The procurement of life-saving organs takes priority before harvesting any VCA; therefore, VCA procedures are considered life-enhancing. However, this issue is still debated among transplantation teams, as prolonged ischemia of any VCA can lead to rejection and affect the long-term survival rate of the graft. Thus, it would be preferable to harvest VCAs from the donor before circulatory arrest and simultaneously harvest the VCA, along with the solid organ whenever feasible, after coordinating with the SOT procurement team to reduce the ischemia time of the grafts [32, 33]. From the perspective of donor site-related issues, the pubic area is not visible during the funeral ceremony, offering a significant advantage. The hidden donor site avoids visible deformities and eliminates the necessity for replacing it with a silicone prosthesis [28].

Immunology-Related Issues

Immunology-related issues pose significant limitations for all VCAs, including penile transplantation, which necessitates lifelong immunosuppression for graft survival and to prevent acute and chronic rejection. The ideal immunosuppressive treatment regimen remains imprecise for many VCA applications, including penile transplantation. There is ample evidence suggesting that an improper immunosuppressive regimen may impact the long-term functional outcome of the transplanted penis [34]. As a potential advantage, a topical immunosuppressant may be useful in the management of penile transplants, as they consist of skin and mucosa [35], but this needs to be evaluated in animal studies. In their ex vivo model of human penile transplantation and rejection, Sopko et al. found that FK506 might be a better-suited immunosuppressant in penile allotransplantation [36].

In clinical follow-up of VCA, it is crucial to detect potential rejection episodes as early as possible, necessitating routine or for-cause skin biopsies for histologic evaluation and grading of rejection [37]. However, skin biopsies may trigger inflammation and rejection, which can lead to further damage to the allograft. A challenge in penile transplantation is the limited skin area available for biopsy. Therefore, sentinel flaps may be required to monitor the immunologic status of the allograft [38]. Additionally, grading and the appearance of rejection may differ from other VCAs, as the penile shaft skin is hairless, highly elastic, and lacks subcuticular fat, distinguishing it from other parts of the body. This requires the establishment of rejection grading criteria specific to penile transplantation, which must be established through preclinical studies.

Chronic rejection, leading to vascular obliteration and graft failure, poses a significant challenge to penile transplantation as well [39, 40]. This can result in erectile dysfunction despite a viable graft in the long term. Furthermore, repetitive trauma from sexual activity may also trigger and potentially increase the risk of rejection episodes [23].

Rescue procedures serve as the primary backup plans when unavoidable rejection attacks occur or the patient's general medical condition does not permit the maintenance of the allograft. In patients undergoing face transplants, rescue procedures and the subsequent reconstruction ladder are more complex compared to those in hand transplant patients. However, in penile transplant patients, all of the aforementioned issues resemble those in hand transplants. Similar to hand transplants, a penile allograft can be easily removed if the survival of VCA is compromised or if life-threatening situations arise. Patients must be informed that the allograft may be removed if the benefit/risk ratio decreases [28].

Functional Issues

Two essential functions of the penis are voiding and erection. Urethral stricture poses a major problem in penile reconstruction [41]. The rate of urethral complications in penile replantation and reconstruction ranges from around 6.7% to up to 68% [28, 42-44]. Early findings of transplantation show promising results regarding urethral function. However, the effects of several acute rejection episodes or chronic rejection on urethral tissue are not known. It is plausible to assume that these conditions may trigger fibrosis and lead to severe urethral stricture in the long term.

Functional erection is one of the important aspects of penile transplantation [45]. Erectile function in two clinical cases of penile transplantation is quite satisfying, but these findings are preliminary, and the quality of the erection is not measured [3, 7]. Erection in the long term remains unknown. Complications such as diabetes mellitus or chronic rejection affecting the vascular tree may cause secondary impotence, which may need to be addressed by medical [46], stem-cell therapy [47-62], or surgical treatment. However, surgical interventions like the insertion of a surgical prosthesis may carry additional risks of infection and possible rejection.

Animal models play a crucial role in understanding erectile physiology. Both small and large animal models are being utilized for studying erectile dysfunction related to novel drug discovery, including diabetic, castration, smoking, and hypercholesterolemia models [63]. Stem cell therapy for erectile dysfunction has shown promise in animal models [50, 52, 53, 57, 61, 64-77] and clinical trials [60, 78-85]; however, its routine clinical translation requires further study. Additionally, small and large animal models of cavernous nerve reconstruction to restore erectile function have been investigated [86-94]. However, the presence of an os penis, which provides structural reinforcement to the penis in canines and some non-human primates (NHP), limits the translatability of canine models to human research [45]. Furthermore, these large animal models have higher husbandry costs and some ethical considerations. Rodents are the most commonly used small animal models for erectile dysfunction studies due to their advantages in handling, low-cost husbandry, and genetic manipulation. Nevertheless, their translation to human studies has numerous limitations and disadvantages [45, 63]. Despite the numerous experimental animal models for erectile dysfunction research, there are currently no established models to address potential erectile dysfunction following penile allotransplantation.

Psychological and Social Issues

Patients undergoing penile transplantation face numerous significant psychological challenges both before and after the procedure. Before transplantation, the psychological issues are confined to the emotional burden of living with a penile defect, such as amputation, which can lead to severe distress. Merwe et al., who performed two cases of penile transplantation, documented the profound psychological impact of penile loss on individuals, emphasizing the devastating effects on self-esteem and identity. Both participants expressed severe depression, at times contemplating suicide, feeling cast out, worthless, and ashamed [8]. After transplantation, a wide array of psychological issues arises due to the unique complexities of penile transplantation. Acceptance of the donor's penis is crucial, requiring psychological readiness from both the recipient and their partner [6, 28, 95-98]. It's essential to consider the mental well-being of the patient's partner before and after the surgery, as the partner's acceptance of the graft is just as crucial as the patient's. The potential consequence is the premature removal of the transplant, as demonstrated by the failure of the initial penile transplant in China. The transplant was withdrawn on day 14 due to the psychological distress experienced by both the recipient and his partner [4, 5]. Patients considering penile transplantation should also possess the psychological resilience to cope with potential complications, failure, or, in the worst case, the removal of the transplant. Routine psychological evaluations are recommended for such candidates, emphasizing the significance of assessing their social support while prioritizing emotional adaptation for both the recipient and their families [97]. Penile transplantation presents distinct religious and cultural challenges, especially concerning the donation and acceptance of genital organs. ​The complex religious viewpoints underscore the importance of comprehensive ethical considerations and counseling for both donors and recipients involved in penile transplantation [26, 31].

## Conclusions

From the clinical relevance standpoint, it’s evident that the models described in the literature fall short of addressing clinical challenges as outlined above. While the most clinically relevant model is described in dogs, it still lacks evaluation of immunologic and erectile functional results. Another challenge is the absence of a suitable animal model for translation to penile transplantation research currently. Despite having various experimental animal models for erectile dysfunction research, there are currently no established models to address possible erectile functional issues following penile transplantation. Additionally, the impact of chronic rejection on functional outcomes in penile transplantation remains unknown and requires further investigation, given its effects on both macro and microvasculature. Therefore, ideal animal models are needed to address surgical techniques, post-surgical complications such as urethral fistulas, immunosuppressive regimens, allograft rejection, and long-term functional evaluations like penile erection following penile transplantation. With the development of clinically relevant animal models, long-term success in penile transplantation outcomes can be significantly improved. The major challenge lies in the absence of a suitable animal model for clinical translation in penile transplantation. However, research should continue using currently available animal models to enhance clinical outcomes in this field.
